# Factors associated with prolonged viral shedding in older patients infected with Omicron BA.2.2

**DOI:** 10.3389/fpubh.2022.1087800

**Published:** 2023-01-12

**Authors:** Weijie Zhong, Xiaosheng Yang, Xiufeng Jiang, Zhixin Duan, Wei Wang, Zhaoliang Sun, Wanghao Chen, Wenchuan Zhang, Jie Xu, Juan Cheng, Xiaoling Yuan, Yi Li

**Affiliations:** ^1^Department of Neurosurgery, Ninth People's Hospital, Affiliated to Shanghai Jiao Tong University School of Medicine, Shanghai, China; ^2^Department of Infectious Disease, Shanghai Ninth People's Hospital, Shanghai Jiao Tong University School of Medicine, Shanghai, China; ^3^Department of Ultrasound, Xinhua Hospital, Shanghai Jiao Tong University School of Medicine, Shanghai, China

**Keywords:** viral shedding time, SARS-CoV-2, omicron, vaccination, corticosteroid

## Abstract

**Background:**

This study explores the risk factors associated with viral shedding time in elderly Chinese patients infected with severe acute respiratory syndrome coronavirus 2 (SARS-CoV-2) omicron.

**Methods:**

Participants infected with SARS-CoV-2 omicron were enrolled in a retrospective study, and divided into two groups according to shedding time (≥10 days, “late clearance group” and <10 days, “early clearance group”).

**Results:**

A total of 180 patients were enrolled in the study (88 early, 92 late), with a median viral shedding time of 10 days and a mean age of 77.02 years. Prolonged SARS-CoV-2 omicron shedding was associated with old age (*p* = 0.007), lack of vaccination (*p* = 0.001), delayed admission to hospital after onset of diagnosis (*p* = 0.001), D-dimer (*p* = 0.003), and methylprednisolone treatment (*p* = 0.048). In multivariate analysis, vaccination (OR, 0.319, 95% CI, 0.130–0.786, *p* = 0.013), Paxlovid (OR, 0.259, 95% CI, 0.104–0.643, *p* = 0.004), and time from onset of diagnosis to admission (OR, 1.802, 95% CI, 1.391–2.355, *p* = 0.000) were significantly associated with viral clearance.

**Conclusions:**

Time from onset of diagnosis to hospitalization, lack of treatment with Paxlovid, and lack of vaccination were independent risk factors in elderly Chinese patients infected with SARS-CoV-2 omicron for prolonged viral shedding.

## Introduction

Coronavirus disease 2019 (COVID-19) was identified in January 2020 and since this time has been the cause of global human-to-human transmission (1, 2). Five variants of concern (VOC) have been identified so far, namely Alpha, Beta, Gamma, Delta, and Omicron as designated by the World Health Organization (WHO) (3, 4). The rapid spread of the omicron variant was first identified on 24 November 2021, and it has since become the predominant variant, posing a threat worldwide (5, 6). Compared to DNA viruses, RNA viruses have a higher rate of mutation (7), and Researchers have found that the variant of omicron is the most mutated strain (8, 9) of SARS-CoV-2 variants, which may help the virus evade infection-blocking antibodies (7). These mutations affect the characteristics of SARS-CoV-2 omicron such as infectivity, immune escape, viral shedding time, and outcome. Data have shown that the infectivity of SARS-CoV-2 omicron variants is 10-fold higher than that of alpha, but omicron patients were less likely to be admitted to the hospital and require ICU-level care (10). In addition, reinfection has been observed approximately 10 times more frequently than with the Delta variant (11, 12). Therefore, omicron is expected to impact the therapeutic effect of COVID-19 drugs significantly, as well as immunity secondary to vaccination or prior infection, infectivity, and outcome (13).

In 2022, a wave of COVID-19 rapidly appeared in Shanghai, China, and after comparing the genomes of viruses, researchers found that the genomes of the infected viruses in Shanghai belonged to the Omicron BA.2.2 strain (14, 15). During this period, Zhang et al. found that the total number of patients in Shanghai was much higher than before and that the ratio of severity and mortality was much lower (5). In addition, studies on people infected with omicron variants have shown that age is positively correlated with severity (16, 17).

Understanding the kinetics of infectious viral shedding to possible transmission risk is crucial to guiding infection prevention and control strategies (18). Therefore, it is essential to understand the shedding time of the omicron variant since it is a crucial factor in the guidance of decisions about isolation precautions and antiviral treatment (19, 20). Through a retrospective cohort study that included 59 hospitalized patients with COVID-19, old age was independently associated with long-term virus shedding (21), and another study demonstrated that sex, corticosteroid use, and delayed admission were independent risk factors for prolonged virus shedding time (22) (non-omicron variants). However, whether these findings are also applicable to the omicron variants is still unclear. To attempt to answer this question, we used the search terms (“Omicron”) and (“shedding time”) and (“prolonged”) to search PubMed up until 24 June 2022 and found no relevant articles. In short, the relationship between omicron viral shedding time and risk factors has not been fully clarified. Hence, this study aims to evaluate the characteristics of viral shedding time with older patients infected with omicron in order to identify risk factors that influence the duration of viral shedding.

## Methods

### Patient enrollment

**Figure 1:**
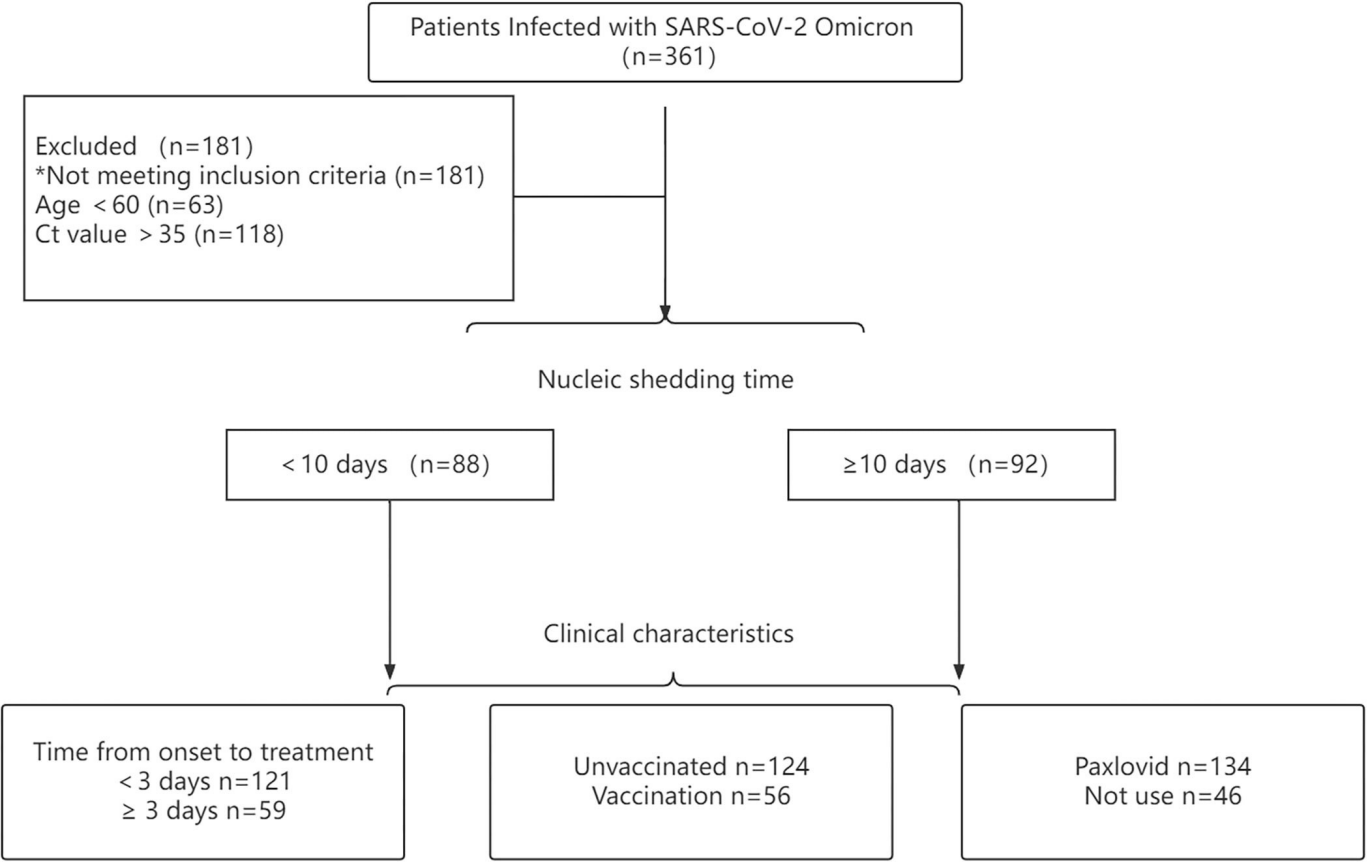
Flow diagram of patients with confirmed SARS-CoV-2 omicron variant included in this study.

All participants were diagnosed with SARS-CoV-2 using real-time PCR following the national guidelines of China (version 9). A total of 361 participants with confirmed SARS-CoV-2 Omicron BA.2.2 admitted to the Ninth People's Hospital Affiliated with Shanghai Jiao Tong University School of Medicine were enrolled for analysis (Figure 1).

According to the characteristics of SARS-CoV-2 omicron, older patients were at greater risk for exacerbation of the disease. Hence, this study aimed to explore the occurrence of viral shedding in older patients infected with omicron variants. Inclusion criteria were as follows: (1) Ct value <35 for both *ORF1ab* and *N* gene and (2) age ≥60. Exclusion criteria were: (1) patients re-infected with SARS-CoV-2 and (2) age <60. According to these criteria, 180 patients were enrolled. Among all patients, the viral shedding time of 88 patients was ≤10 days, and that of 92 patients was ≥10 days.

This trial received approval from the Ethics Committee of the Ninth People's Hospital, Shanghai Jiao Tong University School of Medicine (No. SH9H-2022-T112-2) and was registered at the Chinese Clinical Trial Registry (ChiCTR2200060700).

### Trial procedures

This retrospective cohort study was designed to assess the time of virus shedding in elderly participants infected with SARS-CoV-2 omicron (Figure 1). As such, patients whose viral shedding time was <10 days were classified as the early viral clearance group, and patients whose viral shedding time was ≥10 days were classified as the late viral clearance group. Clinical characteristics and treatment of patients were collected using electronic medical records. The clinical characteristics were as follows: (1) age, mean, (2) cycle threshold value (CT. N and ORF), (3) time from onset of diagnosis to enrollment in patients, (4) gender, (5) condition at admission, (6) vaccine status, (7) comorbidity, (8) first symptoms, (9) laboratory indicators, (10) time from the first admission to the negative testing, and (11) viral shedding time. The treatment of patients consisted of any of the following: (1) medication, (2) auxiliary breathing, and (3) ICU-level care.

### Definitions

Nucleic acid negative tests were recognized as viral shedding (two consecutive, Ct value >35 for the *ORF* and *N gene*) and were quantified by RT-PCR (5). Viral shedding time was defined from the first positive nucleic acid test to the first negative test (in two consecutive, intervals of 24 h) (5). The time from the onset of diagnosis to patient enrollment was considered to be the time from the first positive nucleic acid test to the date of first hospitalization (23, 24).

Conditions at admission included: (1) asymptomatic cases, (2) mild cases, (3) moderate cases, and (4) severe cases, according to Chinese national guidelines (version 9). Mild cases: the clinical symptoms were mild, and no pneumonia was found on imaging. Moderate cases: clinical manifestations and pneumonia could be seen on imaging. Severe cases: progressive aggravation of clinical symptoms, and pulmonary imaging showed that the lesions progressed more than 50% within 24–48 h. Medication was defined as the therapeutic drugs used during hospitalization, including Paxlovid, anticoagulants, methylprednisolone, and traditional Chinese medicine. Assisted breathing included nasal tube oxygen inhalation, mask oxygen inhalation, ventilator oxygen inhalation, and ECMO.

### Statistical analysis

Categorical variables were expressed as numbers (%), and continuous variables were expressed as median (IQR) or mean (standard deviation, SD). Continuous variables were compared using the Mann–Whitney *U*-test or Student's *t*-test, and categorical variables were compared using the χ^2^ test or Fisher's exact tests. Logistic regression was employed to analyze risk factors, and the adjusted odds ratio (OR and 95% CI) was calculated. The values of *p* < 0.05 were considered to indicate statistically significant test results. The different rate of negative nucleic acid tests between groups was compared using the Kaplan–Meier method with a log-rank test.

## Results

### Characteristics of participants in this trial

This study included 180 older participants infected with the omicron variants. Among them, the median time of viral shedding was 10 days, with a mean age of 77.02, and 104 (57.78%) were female. The median time from onset of diagnosis to enrollment was 1 day (1–3 days), and the viral shedding time was 10 days (8–12 days). Most patients were mild cases, and only 1 case (0.56%) was diagnosed as severe. A total of 56 patients (31.11%) were vaccinated, and 124 (68.89%) were unvaccinated. During hospitalization, 174 (96.67%) patients were treated with traditional Chinese medicine, and 134 (74.44%) patients were treated with Paxlovid. In addition, 28 (15.56%) patients needed nasal-catheter-assisted oxygen inhalation during hospitalization.

### Risk factors for viral shedding

The purpose of this study was to observe the virus-shedding time of elderly patients infected with SARS-CoV-2 omicron. To this end, the participants have divided into two groups: one had a viral shedding time <10 days (*n* = 88), and the other had a viral shedding time ≥10 days (*n* = 92). Clinical characteristics, epidemiological features, treatment, laboratory indicators, and outcomes were compared between the two groups (Table 1). No significant differences were found in Ct values or sex. Variables with statistical significance (*p* < 0.05) between the two groups included age (75.05 vs. 78.91, *p* = 0.007), vaccination [38 (43.18%) vs. 18 (19.57%), *p* = 0.001], D-dimer (0.48 vs. 0.97, *p* = 0.030), time from onset of diagnosis to enrollment (1 vs. 3, *p* = 0.001) and time from the first day of admission to testing negative (6.18 vs. 8.7, *p* = 0.001). Compared to the late viral clearance group, the early viral clearance group had more patients treated with Paxlovid [74 (84.09%) vs. 60 (65.22), *p* = 0.004], and the late virus group had more patients treated with methylprednisolone [6 (6.81%) vs. 15 (16.30%), *p* = 0.048]. In addition, the ratio of severe cases at first hospitalization in the late group was higher than in the early group (0 vs. 1.09%), but no significant differences were found in the condition at admission. Moreover, we found that the mean of the Charlson comorbidity index was higher in the late viral clearance group, but this difference was not statistically significant.

**Table 1:** Comparison of clinical characteristics and treatment responses between groups with different viral shedding time.

**Characteristics**	**Total *N* = 180**	**Viral shedding time**		***p*-value**	*p*-value*
		** <10 days *N* = 88**	**≥10 days *N* = 92**		
Age, mean (SD), year	77.02 (9.74)	75.05 (9.73)	78.91 (9.42)	0.007	0.008
CT.N, mean (SD)^a^	28.80 (2.87)	28.50 (2.91)	29.06 (2.82)	0.196	0.254
CT.ORF, mean (SD)	28.18 (3.36)	27.72 (3.43)	28.71 (3.24)	0.047	0.076
Time from onset of diagnosis to enrollment in patients, median (IQR), day	1 (1–3)	1 (1–2)	3 (1–5)	<0.001	<0.001
Time from the first day treatment to the negative testing, mean (SD), day	7.47 (2.65)	6.18 (1.35)	8.7 (2.99)	<0.001	<0.001
**Sex**					
Male, *n* (%)	76 (42.22%)	35 (39.77%)	41 (44.57%)	0.515	–
Female, *n* (%)	104 (57.78%)	53 (60.23%)	51 (55.43%)		
**Condition at admission** ^b^					
Asymptomatic cases, *n* (%)	12 (6.67%)	9 (10.23%)	3 (3.26%)	0.150	–
Mild cases, *n* (%)	142 (78.89%)	67 (76.13%)	75 (81.52%)		
Moderate cases, *n* (%)	25 (13.89%)	12 (13.63%)	13 (14.13%)		
Severe cases, *n* (%)	1 (0.56%)	0 (0.00%)	1 (1.09%)		
**Vaccine**					
Unvaccinated, *n* (%)	124 (68.89%)	50 (56.82%)	74 (80.43%)	<0.001	–
Vaccinated, *n* (%)	56 (31.11%)	38 (43.18%)	18 (19.57%)		
**Comorbidity**					
Hypertension, *n* (%)	110 (61.11%)	53 (60.23%)	57 (61.96%)	0.812	–
Diabetes, *n* (%)	41 (22.78%)	15 (17.05%)	26 (28.26%)	0.073	–
Coronary artery disease, *n* (%)	32 (17.78%)	19 (21.59%)	13 (14.13%)	0.205	–
Charlson, median (IQR)^c^	1 (0–1)	1 (0–1)	1 (0–2)	0.051	0.101
**First symptoms**					
Fever *n* (%)	84 (46.67%)	43 (48.86%)	41 (45.05%)	0.610	–
Fatigue, *n* (%)	45 (25%)	24 (27.27%)	21 (22.83%)	0.491	–
Cough, *n* (%)	141 (78.33%)	66 (75%)	75 (81.52%)	0.288	–
Expectoration, *n* (%)	109 (60.56%)	52 (59.09%)	57 (61.96%)	0.694	–
Runny nose, *n* (%)	56 (31.11%)	24 (27.27%)	32 (34.78%)	0.277	–
Sore throat, *n* (%)	64 (35.56%)	39 (44.32%)	25 (27.17%)	0.016	–
**Laboratory indicators**					
WBC, mean (SD), /L	4.95 (1.48)	4.71 (1.40)	5.18 (1.52)	0.850	0.064
L, mean (SD), /L	1.32 (0.57)	1.25 (0.45)	1.39 (0.65)	0.124	0.163
ALT, median (IQR), U/L	17 (12.25–25)	21.78 (13–27.5)	22.15 (12–25)	0.336	0.230
AST, median (IQR), U/L	26.50 (22–33)	27 (21.5–34)	30.40 (23–33)	0.823	0.547
Prothrombin time, mean (SD), s	10.98 (0.82)	10.98 (0.8)	10.98 (0.84)	0.711	0.841
APTT, mean (SD), s	29.43 (3.20)	29.09 (2.83)	29.76 (3.50)	0.230	0.117
Fibrinogen, mean (SD), g/dl	3.20 (0.78)	3.09 (0.63)	3.30 (0.89)	0.016	0.174
Fibrinogen <2, *n* (%)	29 (16.11%)	9 (10.23%)	20 (21.74%)	0.07	–
D-dimer, mean (SD), mg/L	0.43 (0.26–0.79)	0.48 (0.21–0.64)	0.97 (0.28–0.99)	<0.001	0.003
D-dimer >0.5, *n* (%)	72 (40%)	27 (30.68%)	45 (48.91%)	0.030	–
CRP, mean (SD), mg/L	5.44 (2.28–13.8)	8.49 (2.07–10.94)	13.9 (2.36–15.17)	0.05	0.229
CRP >10, *n* (%)	56 (31.11%)	24 (27.27%)	32 (34.78%)	0.345	–
**Treatment**					
Nasal duct, *n* (%)	28 (15.56%)	12 (13.64%)	16 (17.39%)	0.487	–
Paxlovid, *n* (%)	134 (74.44%)	74 (84.09%)	60 (65.22%)	0.004	–
Anticoagulation, *n* (%)	32 (17.78%)	11 (12.50%)	21 (22.83%)	0.070	–
Methylprednisolone, *n* (%)	21 (11.67%)	6 (6.81%)	15 (16.30%)	0.048	–
Chinese medicine, *n* (%)	174 (96.67%)	86 (97.73%)	88 (95.65%)	0.438	–

^*^Indicated U-test.

^a^Real-time PCR Ct value.

^b^According to WHO criteria.

^c^Charlson comorbidity index.

### Factors associated with shedding time

**Figure 2:**
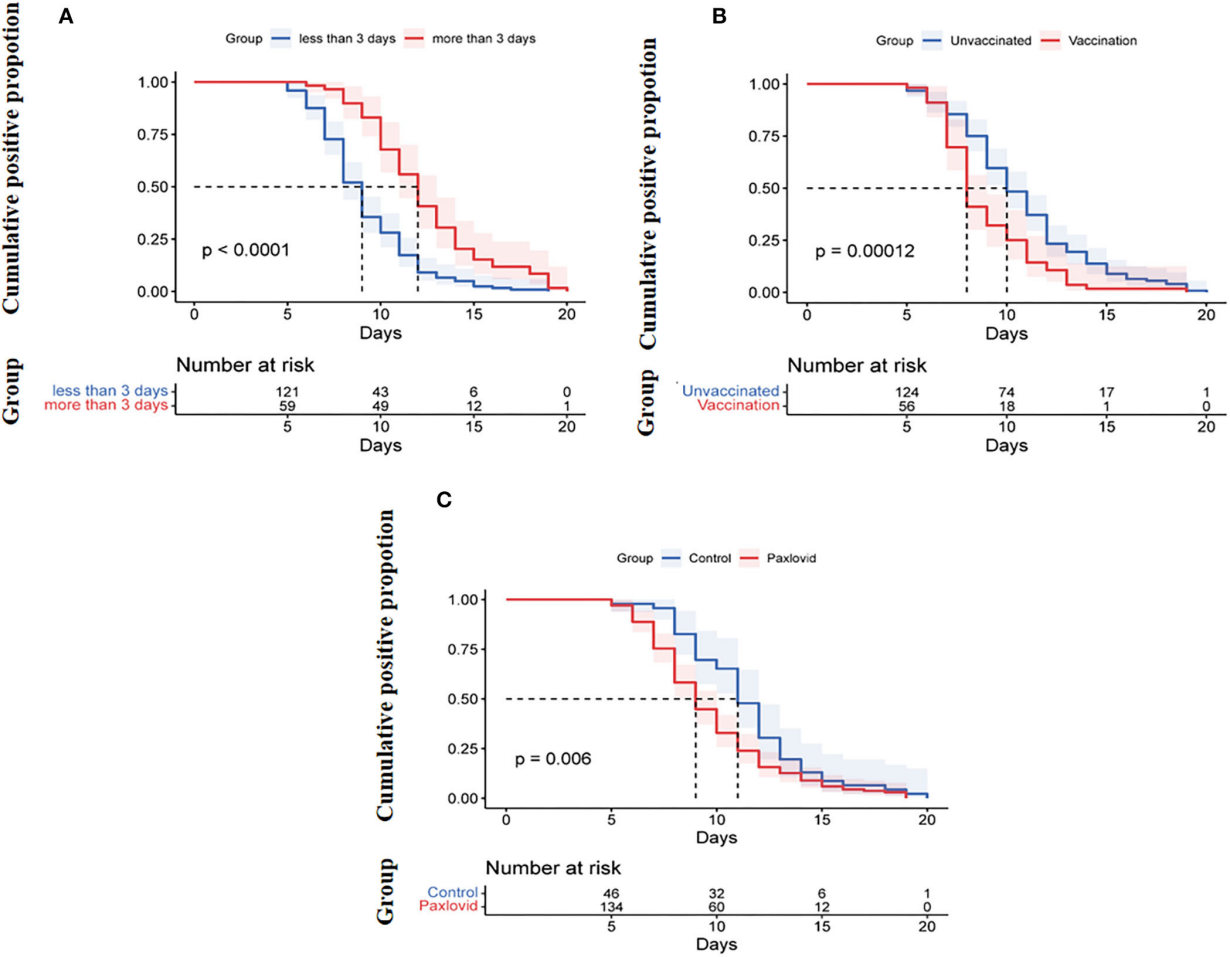
**(A)** Cumulative proportion of the nucleic acid shedding after illness onset by age (log-rank *p* < 0.0001). **(B)** Cumulative proportion of the nucleic acid shedding after the onset of diagnosis between patients admitted to the hospital <3 days and those admitted ≥3 days after onset of diagnosis (log-rank *p* = 0.00012). **(C)** Cumulative proportion of the nucleic acid shedding after illness onset of diagnosis between Paxlovid patients or not (log-rank *p* = 0.006).

Variables with statistical significance (*p* < 0.05) between the two groups, including vaccination status, age, time from onset of diagnosis to enrollment, treatment with Paxlovid or methylprednisolone, D-dimer, and Charlson comorbidity index, were tested. The results showed that vaccination (OR, 0.319, 95% CI, 0.130–0.786, *p* = 0.013), treatment with Paxlovid (OR, 0.259, 95% CI, 0.104–0.643, *p* = 0.004), time from onset of diagnosis to enrollment (OR, 1.802, 95% CI, 1.391–2.355, *p* = 0.000), and D-dimer (OR, 2.005, 95% CI, 0.975–4.121, *p* = 0.059) were independent factors associated with viral shedding time (Table 2). Furthermore, Kaplan–Meier curve analysis indicated the cumulative viral negative proportion was higher in patients admitted to the hospital within 3 days after the first positive nucleic acid test (*p* = 0.0001; Figure 2A), and virus shedding time was shorter in vaccinated compared to unvaccinated patients (*p* = 0.0001, Figure 2B). Finally, SARS-CoV-2 omicron clearance was delayed in participants treated with Paxlovid during hospitalization compared to patients who were not (*p* = 0.006, Figure 2C).

**Table 2:** Multivariable analyses of factors associated with duration of viral shedding time.

**Variable**	**Multivariable analysis Odds ratio (OR)**	**95% CI**	***p*-value**
Age	1.024	0.983–1.067	0.246
Vaccinated	0.319	0.130–0.786	0.013
Time from onset of diagnosis to enrollment in patients' days	1.802	1.391–2.355	0.000
Paxlovid	0.259	0.104–0.643	0.004
Methylprednisolone	2.390	0.713–8.016	0.158
D-dimer	2.005	0.975–4.121	0.059
Charlson	1.288	0.928–1.787	0.130

### Clinical characteristics related to Paxlovid and vaccination status

There were 56 vaccinated patients, and 124 were unvaccinated (Table 3), and we did not find a significant difference in viral shedding time after comparing vaccinated to unvaccinated patients. However, the time from the first day of admission to the negative testing was longer in unvaccinated patients (7.85 vs. 6.62, *p* = 0.004). Additionally, during the time of our study, 134 patients were treated with Paxlovid, and participant characteristics were similar between the treated and untreated Paxlovid groups (Table 3), although Paxlovid was associated with viral shedding time (8.26 vs. 7.19, *p* = 0.018).

**Table 3:** Comparison of clinical characteristics between groups of vaccine status or paxlovid status.

**Parameters**	**Vaccine status**			**Paxlovid**		

	**Unvaccinated** ***N*** = **124**	**Vaccinated** ***N*** = **56**	***p*** **-value**	**Untreated** ***N*** = **46**	**Treated** ***N*** = **134**	***p*** **-value**
Age, mean (SD), year	78.79 (9.73)	73.11 (8.61)	<0.001	77.20 (9.99)	76.96 (9.69)	0.889
**Sex**						
Male, *n* (%)	48 (38.71%)	28 (50%)	0.156	23 (50%)	53 (39.55%)	0.216
Female, *n* (%)	76 (61.29%)	28 (50%)		23 (50%)	81 (60.45%)	
**Condition at admission**						
Asymptomatic cases, *n* (%)	9 (7.26%)	4 (7.14%)	0.696	2 (4.35%)	11 (8.21%)	0.675
Mild cases, *n* (%)	102 (82.26%)	49 (87.50%)		39 (84.78%)	112 (83.58%)	
Moderate cases, *n* (%)	12 (9.68%)	3 (5.36%)		5 (10.87%)	10 (7.46%)	
Severe cases, *n* (%)	1 (0.81%)	0		0	1 (0.75%)	
CT.N, mean (SD)	28.79 (2.94)	28.79 (2.74)	0.996	29.20 (2.73)	28.65 (2.91)	0.259
CT.ORF, mean (SD)	28.32 (3.39)	28.03 (3.32)	0.592	28.79 (3.17)	28.04 (3.41)	0.191
Time from onset of diagnosis to enrollment in patients, median (IQR), day	1 (1–3.25)	1 (1–2)	0.340	2 (1–4)	1 (1–3)	0.169
Charlson, median (IQR)^d^	1 (0–1)	0 (0–1.25)	0.310	1 (0–2)	1 (0–1)	0.843
WBC, mean (SD), /L)	4.93 (1.48)	5 (1.48)	0.796	5.22 (1.57)	4.86 (1.43)	0.151
L, mean (SD), /L	1.30 (0.90–1.70)	1.25 (1–1.60)	0.960	1.30 (1–1.70)	1.20 (0.90–1.60)	0.478
D-dimer, mean (SD), mg/L	0.45 (0.28–0.79)	0.37 (0.21–0.64)	0.092	0.47 (0.27–0.83)	0.42 (0.25–0.75)	0.212
WB <4.0, *n* (%)	34 (27.42%)	14 (25%)	0.734	9 (19.57%)	39 (29.10%)	0.207
L >1.0, *n* (%)	41 (33.06%)	17 (30.36%)	0.719	14 (30.43%)	44 (32.84%)	0.764
D-dimer >0.5, *n* (%)	55 (44.35%)	17 (30.91%)	0.091	21 (45.65%)	51 (38.35%)	0.384
CRP >10, *n* (%)	34 (27.42%)	22 (40%)	0.094	15 (32.61%)	41 (30.83%)	0.822
Viral shedding time, mean (SD), day	10.76 (3.34)	8.95 (2.53)	<0.001	11.48 (3.08)	9.75 (3.15)	0.002
Time from the first day treatment to the negative testing, mean (SD), day	7.85 (2.85)	6.62 (1.92)	0.004	8.26 (2.78)	7.19 (2.56)	0.018

## Discussion

Few studies have been conducted for the viral shedding time of the omicron variant. To the best of our knowledge, this retrospective research is the first of its kind. We found that age, methylprednisolone therapy, longer time from onset of diagnosis to admission, and D-dimer were associated with prolonged viral shedding. Moreover, our results indicated that the time from onset of diagnosis to hospitalization, lack of treatment with Paxlovid, and lack of vaccination were independent risk factors in patients infected with omicron (Table 2).

Many mutation changes have been found in the omicron variant, significantly impacting both immunities secondary to vaccination or prior infection and the efficacy of therapeutic drugs (13). Unvaccinated patients had a longer viral shedding time than vaccinated patients in our study [38 (43.18%) vs. 18 (19.57%)], and this observation may demonstrate that the vaccine plays a role in accelerating the virus shedding of elderly patients infected with omicron. These findings are also consistent with one prospective, observational study that indicated that the vaccine, especially the booster vaccine, remains effective in preventing severe-stage progression and improving prognosis in patients infected with omicron (25). Similarly, Fan et al. (26) proposed that the vaccine can provide effective protection against omicron, although there will still be breakthrough infections. However, our results only show that the vaccine can shorten the viral shedding time in our population. We cannot determine whether it can decrease disease severity or reduce infection rates.

In addition, a study to observe the factors associated with viral shedding among a cohort of COVID-19 patients indicated that male participants had longer viral shedding and more severe symptoms than females infected with COVID-19 (27). Different from these results, we found no difference between the genders and viral shedding time in elderly patients with omicron, and our result is consistent with Bennasrallah et al. (28), who found no significant difference in time to the viral clearance between genders. Among our 180 patients, the mean age was 77.02 years, and we found that older age prolonged the duration of viral shedding (29). In our analysis of complications, we found differences in the Charlson comorbidity index between the early and late viral clearance groups, but there was no significant difference in hypertension, diabetes, or chronic lung disease between them. We speculate that the reason for this phenomenon is that the patients we included were older, and most of them had multiple complications. We also found that a long time from the onset of diagnosis to admission could prolong the duration of viral shedding. In addition, there was no difference in the disease severity and outcome between patients with early vs. late viral shedding. Future prospective studies are needed to confirm whether elderly patients with prolonged viral shedding are not at risk of severe COVID-19 disease and poor outcomes. From the epidemiological perspective, elderly patients with prolonged viral shedding are mainly contributing to the community spread of COVID-19 infection.

The surprising immune evasion ability of the omicron variant may bring many challenges to specific drug treatments (30). Paxlovid, an oral drug, has received the emergency use authorization from the Food and Drug Administration Agency for treating patients with COVID-19, though the efficacy of Paxlovid in elderly patients infected with the omicron variant is still unclear. By analyzing the usage of Paxlovid between the two groups in this study, we found that the early viral clearance group had been treated with Paxlovid more frequently than the late group. Our results also suggest that Paxlovid can reduce the nucleic acid shedding time. Therefore, in the era of omicron variants, our results suggest that Paxlovid can improve the viral shedding time in elderly patients. In summary, the viral shedding time of elderly patients without vaccination is longer than that of the vaccinated patients. In consequence, the results of this study proposed that unvaccinated elderly patients not receiving Paxlovid are contributing to the transmission of Omicron variants in the community as they are expected to have prolonged viral shedding. In the era of Omicron variants, vaccination is still conducive to reducing the risk of virus transmission.

Treatment with methylprednisolone was found to prolong viral shedding time as well, but it was not an independent risk factor. Previous studies have also demonstrated that corticosteroids prolonged viral shedding time in patients with SARS-CoV-2 (31), and one study reported that treatment with low-dose corticosteroids does not reduce viral shedding time (32). Therefore, the immunosuppressive effect of methylprednisolone may indeed lead to the prolonging of viral shedding time. However, this does not contravene the therapeutic effect of methylprednisolone for COVID-19.

There are several limitations of this study. First, this retrospective study is a single-center study with a small sample size that may cause biases in clinical observations. Second, we attempted to explore the risk factors associated with the viral shedding time, but not everyone was diagnosed on the first day. Third, there is no specific distinction in this article as to whether vaccine-vaccinated patients had been induced with a booster, therefore, future studies should aim to resolve this deficiency. Fourth, participants were only patients aged 60 years and above, which could result in potential bias. Thus, the results presented herein only represent this cohort of patients but not all patients infected with omicron. Finally, the best way to confirm viral shedding would have been through viral cultures. In our study, the laboratory of our hospital did not meet the standards of 3 Laboratory of Biological Safety, so the laboratory was unable to cultivate the SARS-CoV-2 Omicron.

## Conclusion

In conclusion, viral shedding time was defined as the time from the first positive nucleic acid test to the date of the first negative test (in two consecutive days) in our study. This study found that time from onset of diagnosis to hospitalization, lack of treatment with Paxlovid, and lack of vaccination were independent risk factors in elderly Chinese patients infected with SARS-CoV-2 omicron for prolonged viral shedding.

## Data availability statement

The original contributions presented in the study are included in the article/supplementary material, further inquiries can be directed to the corresponding authors.

## Ethics statement

This trial received approval from the Ethics Committee of the Ninth People's Hospital, Shanghai Jiao Tong University School of Medicine (No. SH9H-2022-T112-2) and registered at the Chinese Clinical Trial Registry (ChiCTR2200060700). The patients/participants provided their written informed consent to participate in this study.

## Author contributions

WZho, XJ, XYa, ZD, WW, ZS, WZha, JC, XYu, and YL collected the epidemiological and clinical data. XJ and YL were responsible for enrollment and clinical monitoring. XYa, WC, ZD, XJ, and YL were responsible for the distribution and storage of medicines. WZho, JC, WC, XYu, YL, and XJ were responsible for statistical data. XYa, WZha, XJ, XYu, JC, JX, and YL drafted the manuscript. JC and YL were responsible for funding, study conception, design, revising, and submitting the final manuscript. All authors contributed to the article and approved the submitted version.
